# Diamond Disks

**Published:** 1878-10

**Authors:** 


### DIAMOND DISKS.

The diamond disk is one of our most important instruments. The objection that has so often been made to it—"that it would soon wear out"—has little force.

We have a nickel disk that has been in almost daily use for more than a year, and although it does not cut as rapidly as at first, it does sufficiently for most purposes. When they have become worn so that they do not cut as desired, they can be re-charged with the diamond dust, and are in all respects as good as when first made.

Any who have worn disks can send them to Dr, Babcock and have them re-charged for a small sum. So let none be thrown aside as useless.

